# Early suppression of antiviral host response and protocadherins by SARS-CoV-2 Spike protein in THP-1-derived macrophage-like cells

**DOI:** 10.3389/fimmu.2022.999233

**Published:** 2022-10-20

**Authors:** Noémi Miltner, Tamás Richárd Linkner, Viktor Ambrus, Aya S. Al-Muffti, Hala Ahmad, János András Mótyán, Szilvia Benkő, József Tőzsér, Mohamed Mahdi

**Affiliations:** ^1^ Laboratory of Retroviral Biochemistry, Department of Biochemistry and Molecular Biology, Faculty of Medicine, University of Debrecen, Debrecen, Hungary; ^2^ Doctoral School of Molecular Cell and Immune Biology, University of Debrecen, Debrecen, Hungary; ^3^ Laboratory of Inflammation-Physiology, Department of Physiology, Faculty of Medicine, University of Debrecen, Debrecen, Hungary

**Keywords:** SARS-CoV-2, coronavirus, spike protein, transcriptomics, COVID-19

## Abstract

The severe acute respiratory syndrome coronavirus 2 (SARS-CoV-2) is the causative agent of coronavirus disease-19 (COVID-19). The spike protein (S) of SARS-CoV-2 plays a crucial role in mediating viral infectivity; hence, in an extensive effort to curb the pandemic, many urgently approved vaccines rely on the expression of the S protein, aiming to induce a humoral and cellular response to protect against the infection. Given the very limited information about the effects of intracellular expression of the S protein in host cells, we aimed to characterize the early cellular transcriptomic changes induced by expression of the S protein in THP-1-derived macrophage-like cells. Results showed that a wide variety of genes were differentially expressed, products of which are mainly involved in cell adhesion, homeostasis, and most notably, antiviral and immune responses, depicted by significant downregulation of protocadherins and type I alpha interferons (IFNAs). While initially, the levels of IFNAs were higher in the medium of S protein expressing cells, the downregulation observed on the transcriptomic level might have been reflected by no further increase of IFNA cytokines beyond the 5 h time-point, compared to the mock control. Our study highlights the intrinsic pathogenic role of the S protein and sheds some light on the potential drawbacks of its utilization in the context of vaccination strategies.

## Introduction

As of the date of writing this manuscript, according to data from the World Health Organization (WHO), coronavirus disease of 2019 (COVID-19) caused by the severe acute respiratory syndrome coronavirus 2 (SARS-CoV-2) had claimed the lives of over 6.5 million individual worldwide, with more than 614 million infections reported to date. The spike protein (S) of SARS-CoV-2 is a class I, large trimeric fusion glycoprotein that mediates the initial vital contact with host target cells to establish the infection (1, 2). The protein is composed of two functional subunits (S1 and S2). While the S1 comprises the receptor binding domain (RBD) mediating interaction with the host target cellular surface receptor, the S2 mediates fusion between the viral and cellular membranes, similarly to other coronaviruses (1, 3). In the case of SARS-CoV-2 and the related SARS-CoV, the target cell receptor has been identified as the angiotensin-converting enzyme 2 (ACE2) (4), which is expressed in various tissues, primarily by the lung epithelial cells and in the intestine, and to a lesser extent in other tissues as well (5).

It is important to note that while the transmembrane protease serine 2 (TMPRSS2) has been found to facilitate cleavage at the S1/S2 and the S2’ site; hence enhancing fusion and infectivity (6), infection of cells with diminished or no expression of TMPRSS2 may still be accomplished utilizing endocytotic pathways or perhaps, other transmembrane proteases. Therefore, infection is not only limited to TMPRSS2 expressing cells (7, 8).

Given the crucial role of the S protein in mediating infectivity of SARS-CoV-2, vaccines aimed at inducing immunogenicity against the viral protein had been approved and are being widely deployed; most of which are based on the prototypical Wuhan-Hu-1 SARS-CoV-2 S protein sequence, in an effort to curb the pandemic (9). According to the WHO, almost 12.7 billion vaccine doses have been administered to date, with over 4.9 billion individuals fully vaccinated. While the clinical studies have shown a promising efficacy of many of the vaccines in inducing neutralizing antibodies and cellular immune responses against the Wuhan-Hu-1 and some other mutants, their efficacy appears limited in the face of new multi-mutant variants (10, 11), although, while neutralization studies against newly emerging variants are still ongoing, the final verdict is still underway.

Understanding the exact pathomechanism of the S protein and its interaction with host target receptors is of major importance. Despite the S protein of SARS-CoV-2 being the major target for vaccines, effects of the S protein on the cellular physiology are understudied.

Previously, it was reported that ACE2 in alveolar lung epithelium acts as a membrane receptor for cell signal transduction in response to SARS-CoV S protein, activating fibrosis-associated chemokine (C-C motif) ligand 2 expression through the Ras-ERK-AP-1 pathway (12). This finding was also verified recently in the context of SARS-CoV-2, wherein the S protein resulted in the activation of NF-κB and AP-1 transcription factors, promoting IL-6 trans-signaling by activation of angiotensin II receptor signaling in epithelial cells (13, 14). Moreover, this interaction between ACE2 and the S protein was found to trigger translational silencing of viral mRNA *via* DAP-kinase1 signaling pathway in an effort to perhaps relieve ER stress and unfolded protein response that is detrimental to viral replication (15). S protein of the Middle East respiratory syndrome coronavirus (MERS-CoV) was found to suppress macrophage responses, *via* the induction of interleukin-1 receptor associated kinase M (IRAK-M); a negative regulator of Toll-like receptors (TLR) signaling, in addition to induction of the transcriptional repressor peroxisome proliferator-activated receptor-γ (PPARγ) (16).

On the other hand, S protein was found to increase apoptosis, generation of reactive oxygen species, and intracellular calcium expression in macrophage-like cells, polarizing these cells towards a pro-inflammatory profile (17). Additionally, it was found to affect hematopoiesis and myeloid differentiation *in vitro*, and had a noticeable physiologic impact on peripheral blood cells *ex vivo* (18). A conserved 8-residues sequence (KWPWY/WVWL) in the S protein was found to be similar to a sequence typical in proteins involved in coagulation processes; such as von Willebrand factor, coagulation factor X, fibronectin and Notch (19). According to a study using scanning electron (SEM) and fluorescence microscopy as well as mass spectrometry (MS), S protein was found to directly interact with platelets and fibrin(ogen), which may perhaps contribute to the hypercoagulability state observed in COVID-19 (20).

Monocytes and monocyte-derived macrophages (MDMs) play a fundamental role in adaptive immunity against viral infection (21). Upon stimulation, they acquire inflammatory effector functions and aid in antigen-presentation and tissue repair (22). Given their expression of the ACE2 receptor, macrophages were found to be highly susceptible to SARS-CoV-2 infection (23), albeit recent studies have found that infection of monocytes and MDMs by SARS-CoV-2 is abortive, and the pathophysiology is mostly related to stimulation of the production of immunoregulatory cytokines (24). Nevertheless, there is emerging evidence from bulk as well as single cell transcriptomic profiling suggesting that alveolar macrophages from COVID-19 patients can be infected by SARS-CoV-2, resulting in progressive alveolar inflammation (25).

Following intramuscular injection, inflammatory cells dominated by neutrophils are derived to the site 2-6 hours after the injection, resulting in an extensive phagocytosis of vaccine particles (26). Given the inevitable involvement of tissue macrophages in the phagocytosis of vaccine particles, in this study, we were curious as to what transcriptomic changes may occur in monocyte-derived macrophages 5 hours post-transfection with the SARS-CoV-2 S protein, result of which may perhaps unravel its pathologic role in infection, and provide data on its effects as part of vaccination platforms. While most recent studies have carried out an in-depth analysis of cellular transcriptomic changes in target cells and MDMs in the context of SARS-CoV-2 infection (27, 28), our study focuses on the changes induced only by the S protein, which is more relevant given the majority of currently used vaccine formulations.

## Materials and methods

### Cell culturing

THP-1 human monocyte cell line was obtained from ATCC (TIB-202, USA) and cells were maintained in T-75 flask in 15 ml RPMI-1640 culture medium (Sigma-Aldrich, USA) containing 10% heat-inactivated fetal bovine serum (FBS, Gibco, UK), 1% L-glutamine (Sigma-Aldrich, USA) and 1% penicillin-streptomycin (Sigma-Aldrich, USA) in a humidified incubator with 5% CO_2_ at 37°C.

### Plasmid constructs

A pUC57-amp vector encoding the prototypic Wuhan-Hu-1 SARS-CoV-2 S protein (codon optimized for human expression system, pUC57-2019-nCoV-S human) was obtained from GenScript Biotech (USA). The sequence was then cloned into a pcDNA3.1(+)-N-EGFP plasmid (GenScript Biotech, USA) using KpnI and ApaI restriction enzymes and T4 DNA ligase (NEB, UK). The resulting plasmid coded for the SARS-CoV-2 S protein fused to an N-terminal enhanced green fluorescent protein (EGFP) tag, the success of cloning was verified by agarose gel electrophoresis and PCR sequencing (Eurofins Genomics, Germany).

### Activation of THP-1 cells

On the day of differentiation, THP-1 monocytes were plated onto 6-well plate (500.000 cells/well) in 2 ml RPMI-1640 culture medium supplemented with 10% FBS and 1% L-glutamine without antibiotics. After incubation for 3 hours, THP-1 monocytes were activated by 50 nM phorbol-12-myristate-13-acetate (PMA, Sigma-Aldrich, USA) for 1 hour, thereafter, PMA-containing medium was removed carefully, and the cells were incubated in fresh 2 ml antibiotic-free culture medium supplemented with 10% FBS, 1% L-glutamine for a further 24 hours.

### Transfection of differentiated THP-1-derived macrophage-like cells

Macrophage-like cellswere transfected with 2.5 µg of pcDNA3.1(+)-N-EGFP (mock control) or pcDNA3.1(+)-N-EGFP-S plasmids for 5 hours using Lipofectamine reagent (LTX) following the manufacturer’s protocol (Thermo Fisher Scientific, USA). Transfection control cells were only treated with LTX and native, non-transfected but *in vitro*-macrophage-like cells were prepared by replacing the medium with fresh one.

### Determination of SARS-CoV-2 Spike protein expression in transfected cells

To verify expression of the GFP-Spike fusion protein in transfected cells 5 hours after transfection, the cells were washed twice with PBS and re-suspended in lysis buffer (50 mM Tris-HCl, 250 mM NaCl, 5 mM EDTA, 50 mM NaF, 0.5% NP-40, pH=7.4). Thereafter, the cell lysate was incubated on ice for 30 minutes and vortexed every 10 minutes. After incubation, the samples were sonicated (Realsonic Cleaner) and then centrifuged (11,000 g, 4°C, 30 min). The supernatant was transferred into new Eppendorf tubes and Human SARS-CoV-2 RBD ELISA Kit (Invitrogen, USA) was used to determine the amount of SARS-CoV-2 Spike, according to the manufacturer’s instruction.

### Analysis of key pro-inflammatory cytokines by ELISA

5 hours after transfection, supernatants were collected, centrifuged and stored at -20°C until use for cytokine measurements. Cytokine levels of supernatants (IL-1β, TNFα, and IL-6) were determined by using ELISA kits (BD Biosciences, San Diego, CA, USA) according to the manufacturer’s instructions. Quantifications were performed by a FlexStation 3 Microplate Reader (Molecular Devices, Sunnyvale, CA, USA). The detection limits were 0.8 pg/ml for IL-1β, 2 pg/ml for TNFα and 2.2 pg/ml for IL-6. Additionally, levels of interferon alphas (IFNAs) was also determined from the medium at 2h, 4h, 5h, and 24h time-points, with the aid of Human IFN-Alpha ELISA Kit (TCM) (PBL Assay Science, NJ., USA). This time-point analysis was carried out on medium collected from cells transfected in 48-well plate (35,000 cells/well), utilizing 300 ng of plasmids for transfection, otherwise, all the steps of cell activation and transfection were similar to the ones mentioned above.

### RNA isolation, preparation of library, and transcriptome sequencing

5 hours after transfection, the media was removed carefully and cells were washed with 500 µl PBS and homogenized in 500 µl TRIzol Reagent (Molecular Research Center, USA), then the RNA isolation was carried out following the manufacturer’s protocol. Agilent RNA 6000 Nano kit on Bioanalyzer 2100 (Agilent Technologies, Germany) was used to determine the quality and integrity of RNA. Thereafter, high-throughput sequencing was performed on MGI DNBSEQ G400 sequencer (MGI Tech Co., Ltd., China) using MGI Easy RNA Library Prep Set by the Genomic Medicine and Bioinformatics Core Facility of University of Debrecen (Hungary).

### Analysis of transcriptomic data

Sequence quality was determined with FastQC (29). Trimming of adapter sequences and low quality reads was performed with Trimmomatic (version 0.36) (30). Fastq files were then aligned with HISAT2 (version 2.2.1) aligned against the GRCh38 version of the human reference sequence (31). After alignment, the sequences of the samples were quantified with featureCounts (version 1.6.2) (32). Principal component analysis (PCA) was performed and visualized by selecting the top two principal components to demonstrate that the samples are transcriptomically distinct populations. Differential gene expression analysis was carried out with DESeq2 R package (version 1.34.0) applying ‘ashr’ effect size estimator (33). The heatmap for expression profiling of DEGs was generated using pheatmap R package. Gene set enrichment analysis (GSEA) was performed using g:Profiler (34) to identify the biological processes (Gene Ontology BP) associated with the identified genes (p<0.05). We have applied Cytoscape (version 3.9.0) (35) to visualize the DEGs enrichment in various processes.

## Results

In order to carry out our analysis, we have utilized a pcDNA3.1(+) plasmid coding for the SARS-CoV-2 S protein (pcDNA3.1(+)-N-EGFP-S) or the pcDNA3.1(+)-N-EGFP plasmid lacking insert as mock for transfection of the macrophage-like cells. Five hours post-transfection of the cells, total RNA was isolated from cells. In addition, total RNA was also isolated from cells that were treated with Lipofectamine (LTX) reagent without plasmid, and from non-transfected macrophage-like cells, as well. Expression of the S protein was verified by ELISA, and the concentration was found to be 40-70 pg/ml in the lysate of cells transfected with the GFP-Spike fusion protein-coding construct.

### Alteration of genome expression profile by SARS-CoV-2 S

Overall, 2394 significantly altered transcripts were detected in mock-transfected macrophage-like cells, compared to those that were treated only with the LTX reagent. Similarly, significant alterations were detected between S protein and mock-transfected cells, compared to the LTX-treated ones. A full list of differentially expressed genes (DEGs) can be found in **Supplementary Table S1** and **Supplementary Figure S1**.

**Figure 1:**
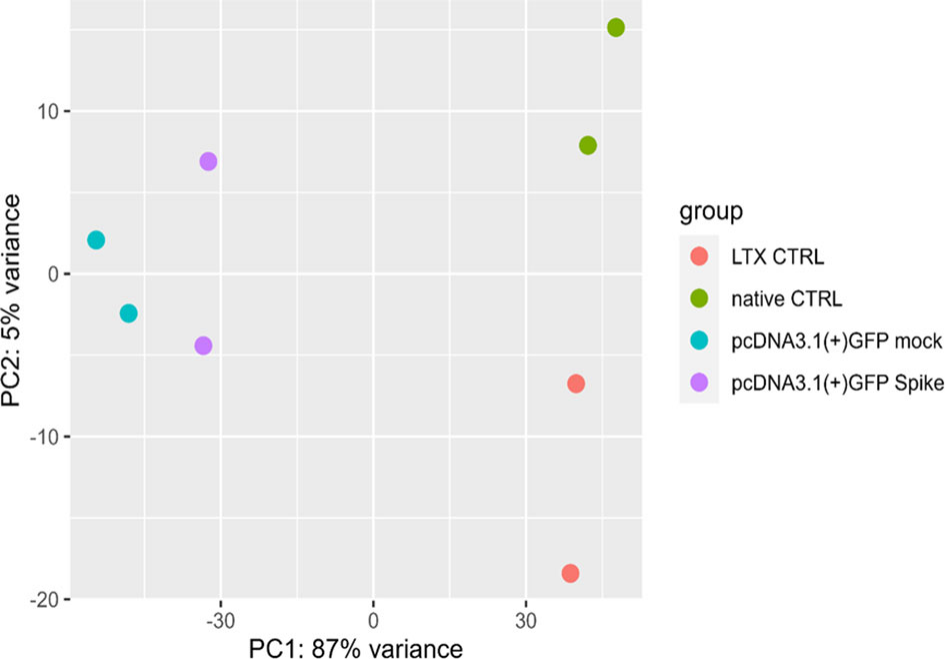
Principal component analysis (PCA). PCA of gene expression profiles in native (untreated) macrophage-like cells control (green, native CTRL), cells treated with only LTX reagent (orange, LTX CTRL), cells transfected with a pcDNA3.1(+)-N-EGFP plasmid (blue, mock) and cells transfected with S protein expressing plasmid (purple).

Statistical analysis was then carried out to identify DEGs in the S protein-expressing cells compared to the mock controls, as depicted by the principal component analysis (PCA) (**Figure 1**).

**Figure 2:**
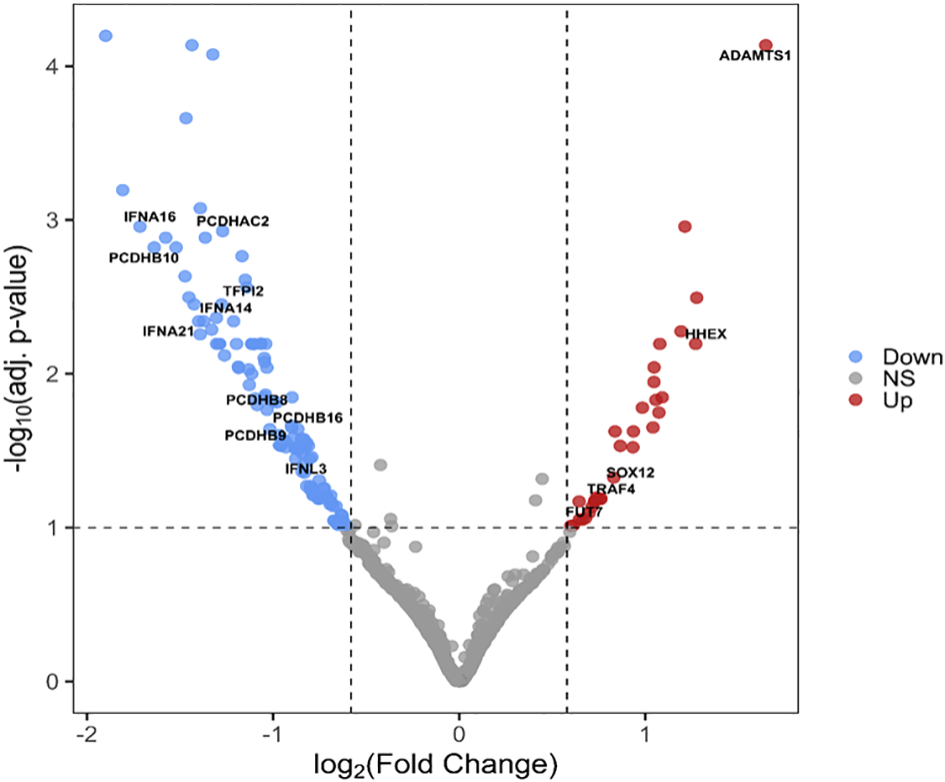
Volcano plot of DEGs between S protein expressing and mock-transfected macrophage-like cells. The -log10 adjusted p-values of the transcripts are plotted as a function of log2(fold change). Red and blue dots indicate the significantly up- and downregulated genes, respectively. Gray dots represent non-significant (NS) transcripts (p> 0.01 or LFC < 0.58 (= FC<1.5)).

We have identified 165 DEGs (adjusted p-value < 0.1, shrunken log_2_(fold change) > 0.58), 142 of which were protein-coding, the most significantly differentially up- and downregulated genes in the S protein-expressing macrophage-like cells 5 hours post-transfection are depicted by the volcano plot in **Figure 2**.

**Figure 3:**
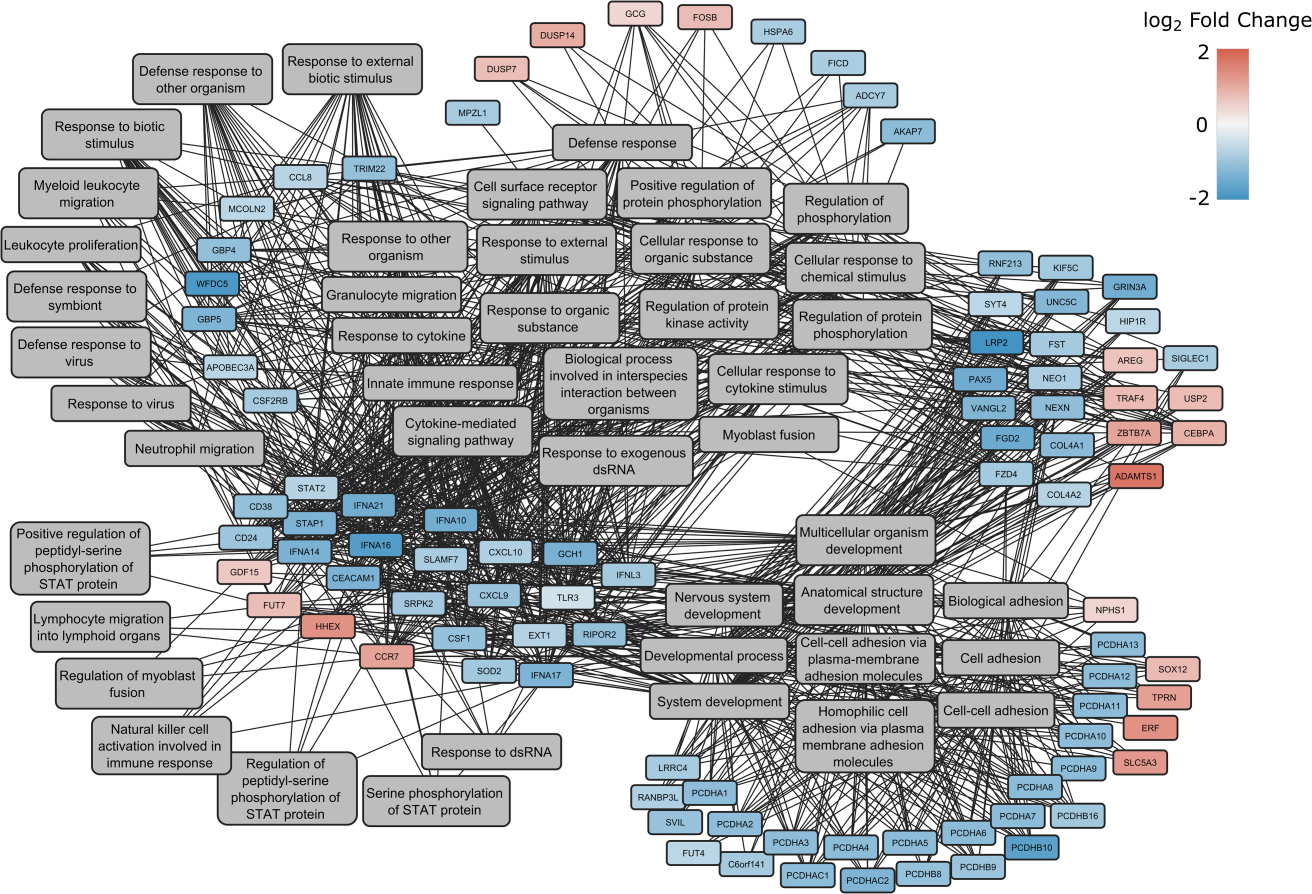
Enrichment network of DEGs between spike-transfected and mock-transfected samples in GO: Biological Processes. The log2(fold change) of the genes are indicated with red and blue colors, while involvement in a process is highlighted with gray lines connecting to the genes with the GO terms.

When classified according to gene ontology (GO) - biological processes (BP), the products of the genes were found to play a role in 12 major processes: multi-organism process, response to stimulus, immune system process, locomotion, cell proliferation, localization, cellular process, multicellular organismal process, biological regulation, biological adhesion, developmental process and metabolic process (**Figure 3**). A comprehensive list of the detected GO terms can be found in **Supplementary Table S1**.

**Figure 4:**
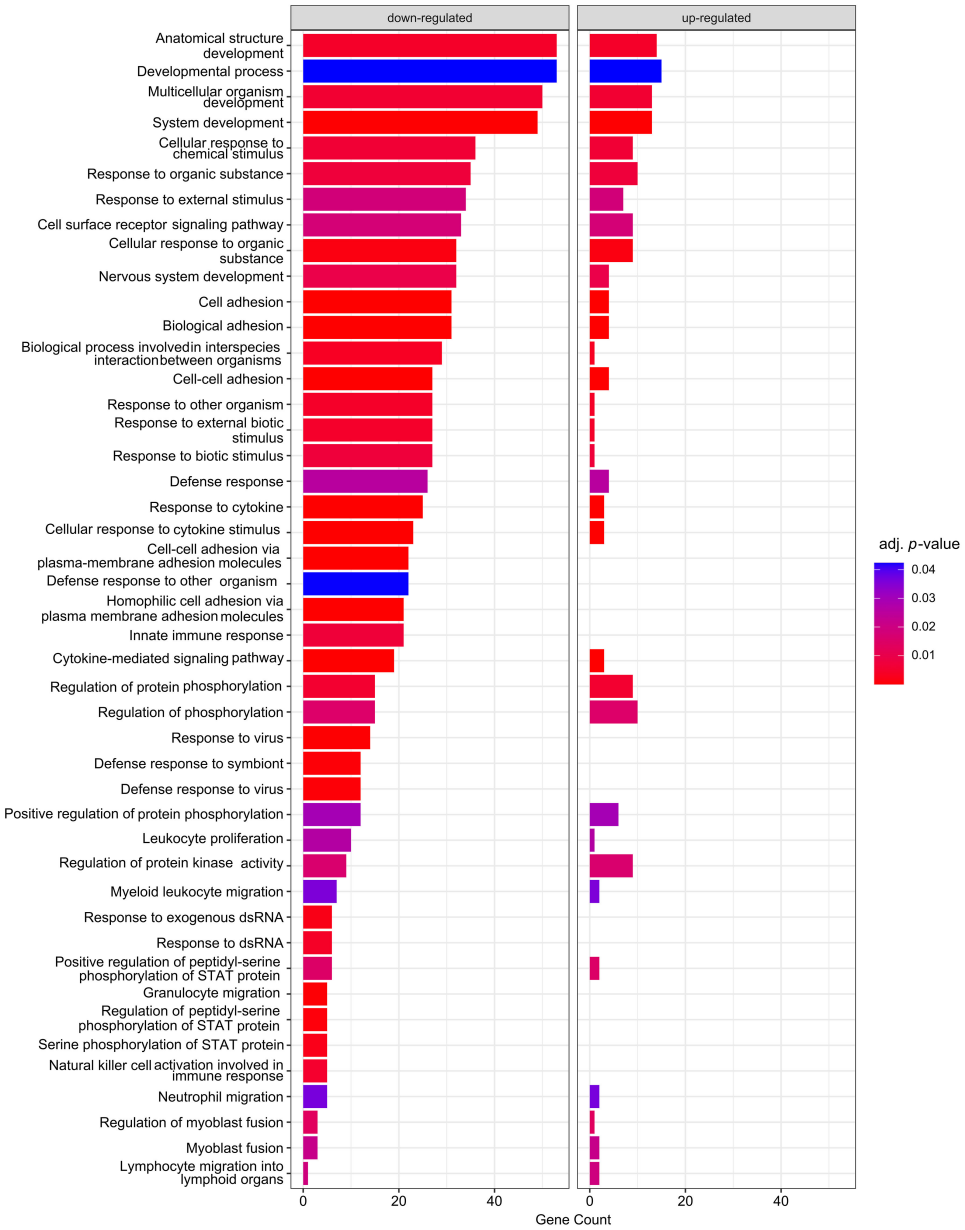
Enrichment analysis of DEGs according to GO: BPs. Analysis was carried out by comparison of S protein–expressing macrophage-like cells to the mock control. Size of the bars represent the genes enriched in a given term, and the colour corresponds of the adjusted p-value of the enrichment.

Next, GO analysis was performed on DEGs induced by the S protein in comparison to the mock control (**Figure 4**).

### Initial dampening of innate immune responses, among others by the S protein

**Figure 5:**
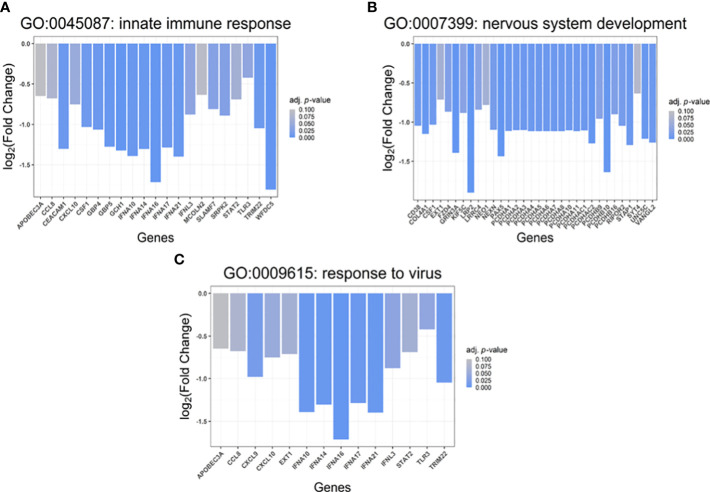
Gene set enrichment analysis (GSEA). Biological processes (Gene Ontology, GO: BP) associated with the identified, differentially up- and downregulated genes (p<0.05) in cells transfected with S protein-coding construct compared to mock control. **(A)** Genes involved in Innate immune response (GO:0045087), **(B)** Genes involved in “nervous system development” (GO:0007399), **(C)** Genes involved in “response to virus” (GO:0009615). The X-axis shows the names of the involved genes while the Y-axis denotes the log2(fold change) of the transcripts.

In total, 21 differentially downregulated genes were found to belong to the innate immune response (GO:0045087) group including interferon alpha isoforms (IFNAs, IFN-alpha 10, 14, 16, 17 and 21), interferon lambda 3 (IFNL3), signal transducer and activator of transcription 2 (STAT2), Toll-like receptor 3 (TLR3), tripartite motif-containing 22 (TRIM22), C-C motif chemokine 8 (CCL8), C-X-C motif chemokine 10 (CXCL10), apolipoprotein B MRNA editing enzyme catalytic subunit 3A (APOBEC3A), carcinoembryonic antigen-related cell adhesion molecule 1 (CEACAM1), SLAM family member 7 (SLAMF7), WAP four-disulfide core domain protein 5 (WFDC5), guanylate-binding protein 4 and 5 (GBP4 and GBP5), colony stimulation factor 1 (CSF1), GTP cyclohydrolase 1 (GCH1), mucolipin TRP cation channel 2 (MCOLN2), and SRSF protein kinase 2 (SRPK2) (**Figure 5A**).

Moreover, 32 down-regulated genes were detected belonging to the nervous system development (GO:0007399) group (**Figure 5B**), including protocadherin alphas 1-11 (PCDHA 1-11), PCDHA subfamily C1-2 (PCDHAC1-C2), protocadherin betas 9, 10 and 16 (PCDHB9/10/16), LDL receptor-related protein 2 (LRP2), CD38 molecule, neogenin (NEO1), leucine-rich repeat-containing 4 (LRRC4), frizzled family receptor 4 (FZD4), paired box 5 (PAX5), synaptotagmin 4 (SYT4), neogenin 1 (NEO1), glutamate ionotropic receptor NMDA type subunit 3A (GRIN3A) as well as nexilin (F-actin-binding protein, NEXN). On the other hand, amphiregulin (AREG), solute carrier family 5 member 3 (SLC5A3), SRY-box transcription factor 12 (SOX12), pseudogene similar to part of protein tyrosine phosphatase receptor type N (PTPRN) were found to be up-regulated in the S protein-expressing cells.

5 hours after transfection with the pcDNA3.1(+)-N-EGFP-S plasmid, we observed downregulation of 14 genes whose products play a crucial role in the antiviral response (GO:0009615); such as IFNA 10, 14, 16, 17 and 21, IFNL3, TLR3, TRIM22, CCL8, C-X-C motif chemokine 9 (CXCL9), CXCL10, glycosyltransferase exostosin 1 (EXT1), APOBEC3A, and STAT2 (**Figure 5C**).

### Early changes in the cytokine profile induced by the S protein

**Figure 6:**
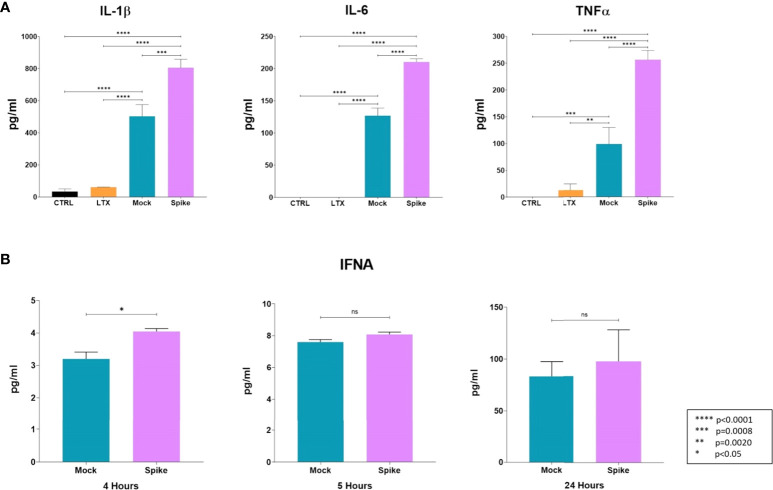
Early changes in the cytokine profile induced by the S protein. **(A)** Protein levels of IL-1β, IL-6, and TNF-α in medium of transfected cells. Results are concluded from triplicate measurements. **(B)** Time-point analysis of levels of IFNAs from media of transfected cells. Results are concluded form duplicate measurements. Error bars indicate SEM. ns, non-significant.

Five hours post-transfection, the cell culture media were collected to analyze changes in the levels of key cytokines (IL-1β, IL-6, TNFα). Transfection with the S protein resulted in a significant elevation of all measured cytokine levels, most notably, level of TNFα was increased more than 2.5 fold compared to the mock control-transfected cells (**Figure 6A**). Moreover, we carried out analysis of IFNAs level in the media of transfected cells at 2 h, 4 h, 5 h, and 24 h time-points. At 2 hours, no IFNAs were detected in the cell culture media of either sample, and increase in IFNA level thereafter, was only detected in the mock control; and the cells transfected with the GFP-Spike fusion protein-coding construct. At 4 h time-point, levels of IFNAs were significantly higher than that of the mock control, thereafter, there was no significant difference in IFNAs level between the two (**Figure 6B**).

## Discussion

In this study, we investigated the transcriptomic changes in THP-1-derived macrophage-like cells induced by the expression of SARS-CoV-2 S protein, the gene expression profile was investigated 5 hours post-transfection. THP-1 is an acute monocytic leukemia cell line, that has long been considered suitable substitutes for human monocytes, with similar viability, cytokine profile and host response (36–38). Activation with PMA induces their differentiation into macrophage-like phenotype, mimicking primary human macrophages (39). Being an immortalized malignant cell line, their response to stimuli may not completely overlap with that of their primary counterparts (37), and some have found significant differences at the level of chromatin structure and topologically associated domains between the two (40). However, the homogeneity of the genetic background of the THP-1 cells (41) minimizes variability. This was advantageous for the purpose of this study, since the presence of variability precipitated by age, gender, and previous infections in peripheral blood mononuclear cell (PBMC)-derived macrophages might have hindered our analysis of the intrinsic pathogenic role of the S protein, although, one might argue that such variability is essential to understanding the pathogenicity in the context of infection with SARS-CoV-2 in different populations.

Our results indicate that expression of the S protein in cells induced significant changes in the transcription profiles of genes that are involved in cell adhesion, homeostasis and most notably, antiviral and immune response.

When we classified the detected transcripts according to GO-term biological processes, transcripts of genes that were involved in cell and biological adhesion were most significantly altered. Members of the clustered protocadherins (PCDH) subfamily of the cadherin superfamily are known to play a major role in cell adhesion, through interaction with β1-integrins (42). PCDHA was shown to be mainly expressed in neuronal cells; such as the olfactory bulb, cerebellum, hippocampus and cerebral cortex (43). In mice model, clustered PCDHA was found to be important for sensory integration and short-term memory (44), others also found that their downregulation or knock-out may predispose to learning defects and aberrant distribution of serotonergic projections in the brain (44, 45). Interestingly, disruption of PCDH diverse expression was found to play a major role in olfactory neural circuit assembly in mice (46). In our experiment, cells transfected with S protein-coding construct showed a marked downregulation of PCDHA 1-11, PCDHA superfamily C-isoforms C1-C2 (PCDHAC1, PCDHAC2) as well as PCDHB 9, 10, 16. Upon treatment of human pulmonary microvascular endothelial cells with thrombin, PCDHA members were among the top up-regulated genes (47), hinting to an uncertain significance in the coagulation cascade and haematological diseases (48).

Of major interest was the downregulation of IFNAs (10, 14, 16, 17, and 21), IFNL3, TRIM22, tripartite motif containing 66 (TRIM66), TLR3, CD38 and CXCL9 induced by S protein expression.

Type I interferons; of which IFNAs are major constituents, play a pivotal role in the early host defense against viral infection. Through binding to IFN receptors, they initiate the JAK/STAT signaling pathway and transcription factors of STAT family, resulting in the transcription of interferon-stimulated genes (ISGs) which act directly against the invading virus (49). It is worth noting that while some ISGs possess antiviral properties, others were found to promote viral replication (50). Of the ISGs that were reported to have potent antiviral response, DDX60, GBP1, IFI44L were also downregulated. These were found to suppress the replication of hepatitis C virus (HCV), human papilloma virus (HPV), encephalomyocarditis virus (EMCV) and the vesicular stomatitis virus (VSV) (50, 51).

While some IFNA genes are rapidly induced by viral infection without the need for ongoing protein synthesis, others show delayed induction in response to ongoing viral protein synthesis (52). IFNA 10, 14, 16 and 17 were found to have high anti-proliferative and antiviral effect in cell culture (53). IFNAs also play an important role in the inhibition of viral reactivation. Previous studies have demonstrated that treatment of Epstein-Barr (EBV)-infected cells with type I interferon inhibits reactivation of the virus (54), similar effects were also observed in the case of cytomegalovirus (CMV) and the herpes simplex virus (HSV) (55).

It has been established that SARS-CoV-2 is a weak inducer of type I interferons in cell culture and animal models (56, 57), moreover, viral proteins such as the accessory protein 9b (ORF9b), non-structural proteins (nsp) 1, 13, and 15 were found to evade and antagonize the induction of type I interferons (58–60). Additionally, pretreatment of kidney epithelial Vero E6 and lung cancer Calu3 cell line with IFN-I resulted in substantially attenuated replication of SARS-CoV-2 (61).

Of the transcripts that were significantly upregulated were the pleckstrin homology domain-containing family A member 6 (PLEKHA6), a disintegrin and metalloproteinase with thrombospondin motif (ADAMTS1) and transcription factors; such as hematopoietically-expressed Homeobox protein (HHEX), FosB proto-oncogene (AP-1 transcription factor subunit), FUT7, SOX12 and CCAAT-enhance-binding protein alpha (CEBPA).

ADAMTS1 was shown to remodel the extracellular matrix and has been implicated in angiogenesis, embryogenesis, wound repair, and cancer development (62, 63).

Interestingly, we found that transfection with the S protein encoding plasmid resulted in the up-regulation of FUT7, product of which catalyzes the addition of a fucose in the sialyl Lewis X (sLeX)-characteristic alpha-(1,3) linkage. Viruses, such as the human T-cell leukemia virus type 1 (HTLV-1) were found to induce sLeX synthesis in a similar manner, through up-regulation of FUT7, which contribute immensely to the pathogenesis of the virus (64).

To gain insight into the changes of key cytokines on the protein level induced by transfection with the S protein-coding construct, we carried out analysis of the medium of transfected cells, and determined the concentration of IL-1β, IL-6, TNF-α, and IFNAs. Rather unsurprisingly, S protein resulted in the upregulation of IL-1β, IL-6, and TNFα protein levels, compared to the mock control. It has been previously documented that the S protein induces IL-1β in a dose-dependent manner (65), and elevated levels of IL-6 and TNF-α were also found to accompany induction with the S protein in a mouse model (66). Given the downregulation of IFNA levels by the S protein on the transcriptomic level, we wanted to explore as to what extent that might influence the cytokine levels, therefore, we conducted time analysis of IFNAs protein level at 2, 4, 5, and 24h time-points. Although initially the levels of IFNAs were higher in the medium of S protein expressing cells, there was no significant difference in their levels after the 5h time-point, compared to the mock control.

Correlation between transcriptomic changes and protein levels cannot always be drawn, and while genomic regulation of certain cytokines; such as TNF-α, may closely mirror their protein levels, other cytokines such as IL-6 may not show a similar pattern (67). In regards to IFNAs, the downregulation of 1-1.5 log-fold changes observed on the transcriptomic level might have been reflected by no further increase of IFNA cytokines beyond the 5 h time-point, compared to the mock control.

In conclusion, our study shows that SARS-CoV-2 S induces complex and orchestrated cellular transcriptomic changes in human monocyte-derived macrophages that undoubtedly contribute to the pathogenesis of infection. According to our knowledge, downregulation of protocadherins and type I interferons by the S protein at an early (5 hours) time-point post-transfection is a novel finding, and should be taken into consideration in the context of vaccination strategies, as this may result in the activation of other latent viral infections; such as EBV infection in vaccinated individuals or during the course of COVID-19 (68, 69).

However, our study is not without limitations. First, expression of the S protein by our plasmid was in fusion with and N-terminal EGFP tag, which may have affected the localization of the S protein, although, the significantly altered transcripts were compared to transfection with the GFP-only expressing plasmid utilized as mock. Also, it would be of interest to see whether or not the differential expression of genes persists beyond the 5 hours’ time-point. Moreover, additional studies utilizing proteomic techniques and proximity ligation assays to study intracellular interactions of the S protein would have been advantageous; however, this may perhaps be the subject of a follow-up study.

Another limitation of our study is that polarization into classically activated (M1) and alternatively activated (M2) macrophages was not carried out, which could have provided important information on the distinct alteration of cytokine response induced by the S protein. Recent studies have shown that distinct macrophage phenotypes show differential immune response patterns to infection (70), and M1 and M2 phenotypes showed noticeable difference in uptake, amplification, and release of SARS-CoV-2 (71). This analysis however, was beyond the scope of our current study, and shall be explored in the future. In this study, we analyzed the transcriptomic response and key cytokine levels in PMA-differentiated THP-1 cells. Cluster of differentiation (CD) marker analysis showed that > 50% of the cells were at M0 stage, as they expressed CD11b, but not CD80 or CD206. This is in line with literature, since THP-1 cells apparently do not express CD80 or CD206, neither in resting nor activated state, but they express CD11b upon stimulation with PMA (72). Therefore, we were not able to verify the rest of the phenotypes, and were not able to ascertain or control for the transcriptomic changes and cytokine profile between M2 and M1 phenotypes.

All in all, our results shed light on the complex pathophysiology of SARS-CoV-2, focusing on the S protein given its wide utilization in vaccination strategies, and despite the current limitations of this study, the findings presented are nevertheless important, providing an insight into how the S protein alters the cellular milieu and transcriptomic profile, in order to establish an optimal cellular environment for viral infection and production.

## Data availability statement

Data has been submitted to GEO repository under GSE208320 identifier.

## Author contributions

Conceptualization: MM, JT. Methodology: MM, NM, AA, HA, and TL. Software: VA. Validation: VA, MM, JT. Formal Analysis: MM, JT, NM and TL. Investigation: MM, NM, AA, HA, TL and VA. Resources: JT, SB. Data Curation: VA. Writing – original draft preparation: MM, NM, TL, VA. Writing – review and editing: MM, NM, JM, JT, SB. Visualization: MM, JT. Supervision: MM. Project administration: JT. Funding Acquisition: JT. All authors contributed to the article and approved the submitted version.

## Funding

Project no. TKP2021-EGA-20 (Biotechnology) has been implemented with the support provided from the National Research, Development and Innovation Fund of Hungary, financed under the TKP2021-EGA funding scheme. This project was supported by the grant for high-risk research in the post-COVID field of the Hungarian Academy of Sciences (POST-COVID2021-16), and by the Hungarian National Scientific Research Fund (NKFIH-OTKA Grant No. K131844) to SB. HA and AA hold a Stipendium Hungaricum Scholarship from the Government of Hungary.

## Acknowledgments

The authors are grateful to the members of Laboratory of Retroviral Biochemistry research group at the Department of Biochemistry and Molecular Biology (University of Debrecen). The authors are grateful for the high-throughput sequencing service of Genomic Medicine and Bioinformatics Core Facility of University of Debrecen (Hungary).

## Conflict of interest

The authors declare that the research was conducted in the absence of any commercial or financial relationships that could be construed as a potential conflict of interest.

## Publisher’s note

All claims expressed in this article are solely those of the authors and do not necessarily represent those of their affiliated organizations, or those of the publisher, the editors and the reviewers. Any product that may be evaluated in this article, or claim that may be made by its manufacturer, is not guaranteed or endorsed by the publisher.

## Supplementary material

Supplementary Table 1 List of GO enrichment terms of RNA transcripts detected from control, mock and spike-transfected THP1 cells.

Supplementary Figure 1 Cluster heatmap of differently regulated genes among the spike-transfected and mock samples.
