# Supplementary figures and images for: Early suppression of antiviral host response and protocadherins by SARS-CoV-2 Spike protein in THP-1-derived macrophage-like cells
Source: Front Immunol. 2022 Oct 20;13:999233. doi: 10.3389/fimmu.2022.999233 (PMC9634736; doi:10.3389/fimmu.2022.999233)

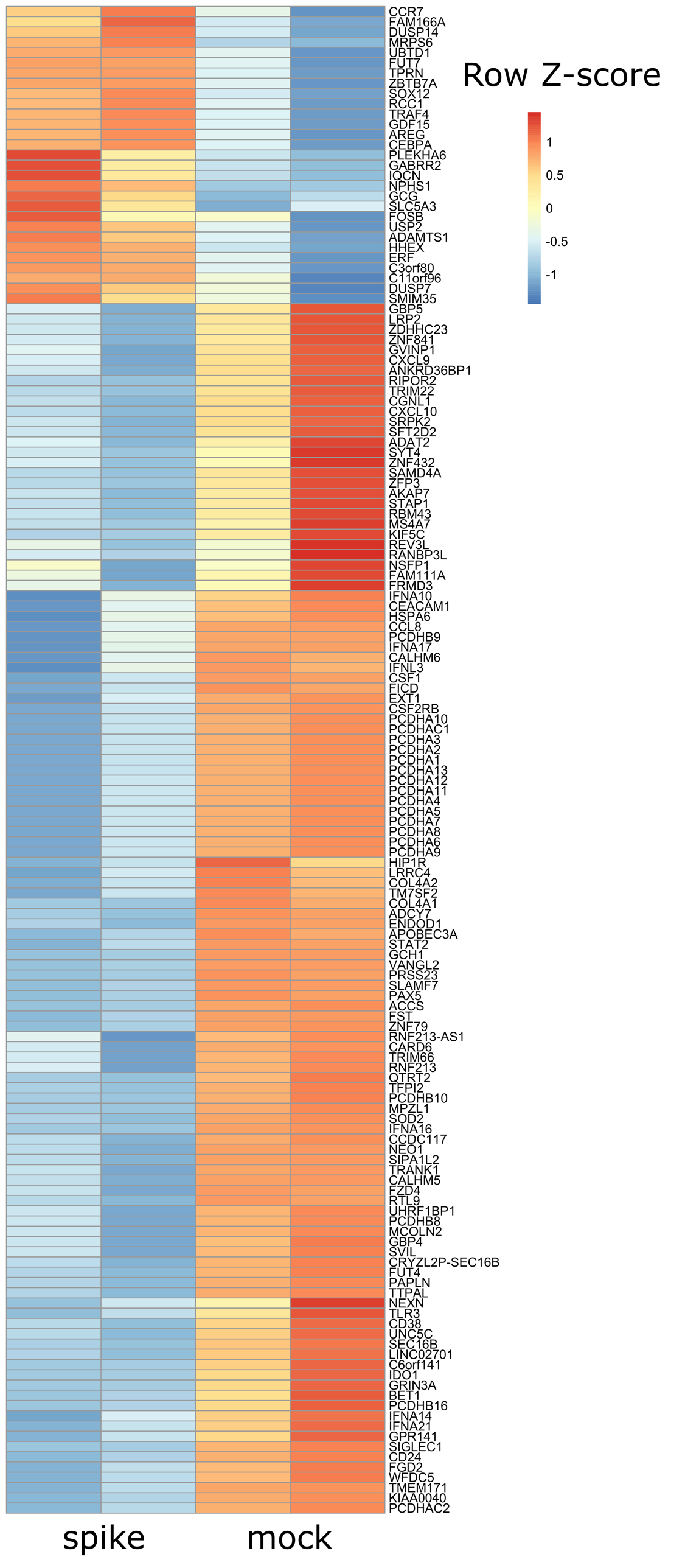

Supplement: Supplementary Figure 1 — Cluster heatmap of differently regulated genes among the spike-transfected and mock samples. [file Image_1.png]
